# Can the WHO ‘s recommendations of physical activity volume decrease the risk of heart disease in middle and older aged Chinese People: the evidence from a seven year longitudinal survey

**DOI:** 10.1186/s12877-022-03276-0

**Published:** 2022-07-18

**Authors:** Meng Ding, Yanan Zhou, Chengxiang Li, Weipeng Li, Ningxin Jia, Xiaosheng Dong

**Affiliations:** 1 College of Physical Education, Shandong Normal University, 17923 Jingshi Road, Jinan, 250014 China; 2 Department of Sport and Health, School of Physical Education, Shandong University, Jinan, 250061 China

**Keywords:** WHO’s recommendations of physical activity, Heart disease, Longitudinal survey

## Abstract

**Background:**

At present, there is a lack of direct evidence to confirm whether the recommendations of the World Health Organization can play a role in fitness and disease prevention in the Chinese population. Therefore, we aimed to analyse 7-year longitudinal survey data to explore whether the physical activity volume recommended by the World Health Organization can help Chinese middle-aged and elderly people reduce the risk of heart disease.

**Methods:**

Data for the 8327 participants who were finally included in this study were derived from the 7-year data of the China Health and Retirement Longitudinal Study (CHARLS) from 2011 to 2018. The physical activity volume is expressed by the product of physical frequency and duration, and heart disease is screened according to self-reported diagnosis and related treatment. The relationship between different physical activity volume groups and the incidence rate of heart disease was determined by a multivariate Cox proportional hazards regression model.

**Results:**

After adjusting for all covariates, participants meeting the WHO’s recommendations had a 20% lower risk of heart disease than those who did not meet the WHO’s recommendations (HR = 0.80, 95% = 0.68–0.96). Subgroup analysis showed that among the participants meeting the WHO’s recommendations, men (HR = 0.71) had a lower risk of heart disease than women (HR = 0.74); in addition, the risk of heart disease was significantly reduced in participants who were middle-aged (26%), had a normal BMI range (49%), did not have hypertension (24%), did not have hyperlipidaemia (21%) and did not have lung disease (21%). It should be noted that the risk of heart disease was reduced by 72 and 67% in participants with untreated hyperlipidaemia and untreated lung disease, respectively.

**Conclusions:**

This study revealed that meeting the WHO’s recommendations for physical activity volume can reduce the risk of heart disease in middle-aged and older people in China and can also effectively prevent heart disease for people with some common chronic diseases, such as hyperlipidaemia and lung disease. The results showed that physical activity for leisure and exercise had a lower preventive effect on heart disease than physical activity for a job, which may be related to the inappropriate leisure and exercise methods of the participants.

**Trial registration:**

IRB00001052–11015.

## Background

Heart disease, including coronary heart disease, heart attack, congestive heart failure and angina pectoris, is a chronic disease with high morbidity and mortality worldwide [1, 2]. In recent years, the prevalence of heart disease has continued to rise, especially in developing countries [3]. According to statistics, nearly 9 million people died of heart disease worldwide in 2019, accounting for approximately 16% of all deaths. It is estimated that the number of patients dying of heart disease will rise to 23 million by 2030 [4]. Heart disease accounts for approximately 40% of disease-related deaths in China [5]. The serious harm caused by heart disease has led to great economic, political, and social burdens on society and has become a major public health problem [4]. Although significant progress has been made in the detection and treatment of heart disease, little is known about its developmental mechanism and characteristics [2]. Therefore, it is very important to strengthen the research on the prevention of and treatment strategies for heart disease [6].

Previous studies have identified a series of unhealthy individual or social factors related to heart disease (such as smoking) [7, 8]. Studies have shown that smoking induces heart disease through the following pathogenesis: (1) endothelial dysfunction, (2) a prothrombotic effect, (3) inflammation, (4) altered lipid metabolism, (5) an increased demand for myocardial oxygen and blood, and (6) a decreased supply of myocardial blood and oxygen [9]. It should be noted that unhealthy lifestyles such as insufficient physical activity (PA) can lead to metabolic abnormalities such as hypertension, hyperlipidaemia, and hyperglycaemia, which lead to heart disease [10]. Epidemiological studies in recent decades have shown that PA is closely related to the incidence rate of cardiovascular diseases [11], and sedentary people are more likely to develop acute cardiovascular events than those who regularly participate in PA [12]. Therefore, some scholars have identified insufficient PA as a risk factor for heart disease [13].

Many clinical and laboratory studies have confirmed that PA, especially exercise, is a safe and effective intervention to prevent the occurrence and development of heart disease. Short-term exercise can increase heart rate, stroke output and cardiac contractility and improve sympathetic function [14, 15]; regular PA or long-term aerobic exercise can produce physiological hypertrophy of the heart, that is, adaptive remodelling of the heart, which is beneficial to cardiovascular health and is of great significance to the prevention and control of heart disease [14, 16, 17].

To help support populations to achieve the target levels and maintain healthy levels of physical activity and to enable people of all ages and abilities to be physically active and improve their health, the World Health Organization (WHO) recommends that middle-aged and older people should perform 150–300 min of moderate-intensity PA, 75–150 min of vigorous-intensity PA, or some equivalent combination of moderate-intensity and vigorous-intensity aerobic PA per week [18]. At present, studies have verified that using the guide to perform PA can prevent the occurrence of heart disease; however, most of these findings come from European and American populations. Compared with developed countries, developing societies face a hostile cardiovascular environment characterized by changes in diet, exercise, the effects of tobacco, socioeconomic stressors, economic constraints at the national and individual levels, and potential risk exposure to new risk factors [19]. In this regard, it needs to be further verified whether the WHO’s recommendations for PA can offset these risk factors and reduce the risk of heart disease among middle-aged and older people in China.

The main purpose of this study was to explore whether the WHO recommendations for PA can reduce the risk of heart disease among middle-aged and older people in China. We assumed that the PA levels recommended by the WHO can reduce the incidence of heart disease in middle-aged and older people in China.

## Methods

### Data source and generation

The data used in the study come from the China Health and Retirement Longitudinal Survey (CHARLS), a major project hosted by the National Development Research Institute of Peking University, which aims to collect a set of high-quality micro data representing Chinese families and middle-aged and elderly individuals to analyse the problem of population ageing in China. The CHARLS national baseline survey was conducted in 2011, covering 17,000 people in approximately 10,000 households in 150 county-level units and 450 village-level units. These samples are tracked every two or 3 years. The CHARLS questionnaire includes basic personal information, family structure and financial support, health status and function, physical measurements, utilization of medical services and medical insurance, work status, retirement and pension status, income status, consumption status, assets, and the basic situation of the community, among which the health status and function part includes personal PA, chronic diseases, physical function and so on, which could provide corresponding research variables for this study.

There were 17,596 and 19,752 participants in the 2011 wave and 2018 wave, respectively. The health status and function data from the 2011 and 2018 waves were merged according to ID. After the merger, data for 13,348 matching participants were obtained. According to the results of the 2011 questionnaire, 1490 participants had existing heart problems and 473 had stroke and physical disability; these participants were excluded, leaving 11,385 participants. After excluding the participants with any missing variables, such as age, sex, height, weight, drinking status, smoking status, chronic diseases, education level, and PA level, 8327 participants were included in the final analysis.

### Assessment of physical activity

In the questionnaire, the participants were asked three questions (Table 1): first, the participants were asked whether they performed the following activities for at least 10 minutes a week: (1) high-intensity PA (HPA), such as carrying heavy objects, digging, farming, aerobics, and fast cycling; (2) moderate-intensity physical activity (MPA), such as carrying light loads, cycling at normal speed, mopping the floor, doing Taijiquan, and fast walking; and (3) low-intensity physical activity (LPA), such as walking, leisure activities, and sports. Second, the participants were asked “During a usual week, on how many days did you perform HPA/MPA/LPA for at least 10 min?” (range 1–7 days). Third, the participants were asked “How much time did you usually spend performed HPA/MPA/LPA on one of those days?” (< 30 minutes, ≥30 minutes, < 2 hours, ≥2 hours, < 4 hours, ≥4 hours).

**Table 1:** Assessment of Physical Activity

Intensity	Low-intensity physical activity (LPA)			Moderate-intensity physical activity (MPA)		High-intensity physical activity (HPA)	
	Walking, leisure, sports, and so on			Carrying light loads, cycling at normal speed, mopping the floor, doing Taijiquan, fast walking, etc.		Carrying heavy objects, digging, farming, aerobics, fast cycling, etc.	
**Frequency (days/week)**	1	2	3	4	5	6	7
**Duration**	<10 mins	≥10 mins, <30 mins		≥30 mins, <2 hours	≥2 hours, <4 hours	≥4 hours	

To calculate the physical activity volume (PAV), referring to the treatment methods of other scholars [20], we converted the time range to an intermediate value: “ ≥ 10 minutes and < 30 minutes” was converted to 20 minutes, “ ≥ 30 minutes and < 2 hours” to 75 min, “≥ 2 hours and < 4 hours” to 180 min, and “ ≥ 4 hours” to 240 min. The result of the PAV is expressed by the product of the PA frequency (how many days per week) and the duration (how much time per day): Physical activity volume (PAV) = PA frequency × duration.

The World Health Organization (WHO) recommends that, for middle-aged and elderly individuals, moderate-intensity and high-intensity activities should be no less than 150 min and 75 min per week, respectively. In addition, the WHO also proposed that an HPAV is twice as effective as an MPAV [18]; according to this principle, we converted the HPAV of the participants in this study into twice the MPAV to facilitate the calculation of the PAV. The participants were divided into two groups based on their calculated PAV: The insufficient PAV group: the participants in this group had only LPA or less than 150 minutes of weekly MPA or LPA and less than 150 minutes of weekly MPA; and the PAV group meeting the WHO’s recommendations: the participants in this group had weekly MPA that was greater than or equal to 150 minutes. In addition, we divided the participants who met the WHO recommendations for PAV into the following three groups according to the purpose of physical activity: job demands (JDs); entertainment or exercise (EE); and job demands and entertainment or exercise (JDs&EE).

### Outcome variable

#### Identification of heart disease

The participants who suffered from heart disease were determined by self-report or were diagnosed by a doctor. The question on the questionnaire was “Have you been diagnosed with heart attack, coronary heart disease, angina, congestive heart failure, or other heart problems by a doctor?” In addition, the time when the participant was diagnosed with heart disease was obtained through self-report, and the question on the questionnaire was “When was the condition first diagnosed or known by yourself? “.

#### Covariates

Several covariates were included as confounders in the study. The individual variables included age, sex (male or female), and education (senior high school and below, college or higher). Body mass index (BMI) was included as a variable related to health status (lean: BMI < 18.5; normal: 18.5 ≤ BMI < 25; overweight: 25 ≤ BMI < 30; obese: BMI ≥ 30). Life behaviour variables included smoking (current smoking, former smoking, never smoking), and drinking (current drinking, former drinking, never drinking); chronic disease status included hypertension, hyperlipidaemia, diabetes, and lung disease, and the participants with chronic diseases were divided into untreated and treated groups according to their self-reports.

### Data analysis

The baseline characteristics of the study participants are reported by using percentages for categorical variables and the mean ± SE for continuous variables. In addition, we tested for differences among participant characteristics between the 2 PAV groups by using an analysis of variance model for continuous variables and the chi-square test for categorical variables. The hazard ratios and 95% confidence intervals (HRs and 95% CIs) were calculated by Cox proportional hazards models to determine the relationship between the different PAV groups and the incidence rate of heart disease. To evaluate the different potential confounding effects of different covariates on the association between PAV and the incidence rate of heart disease, we established three models. We adjusted for age and sex in Model 1, adjusted for age, sex, smoking status, drinking status, education level, and BMI in Model 2 and adjusted for all the covariates in Model 3. The interaction of potential covariates (age, sex, education level, BMI, smoking status, drinking status, and chronic diseases) with the PAV on the incidence rate of heart disease was tested, and we conducted subgroup analysis. In addition, according to the above, there were 3058 participants with missing data, accounting for 38% of the total data. Therefore, to test the stability of the results, we performed a sensitivity analysis with 11,385 participants after multiple imputations for variables with missing values. We used the Markov chain Monte Carlo imputation method and assumed that all the variables in the imputation model show a multivariate normal distribution of joint effects. All statistical analyses were performed in STATA 14 and SPSS 23, and statistical significance was accepted at *P* < 0.05.

## Results

### Population characteristics

**Fig. 1:**
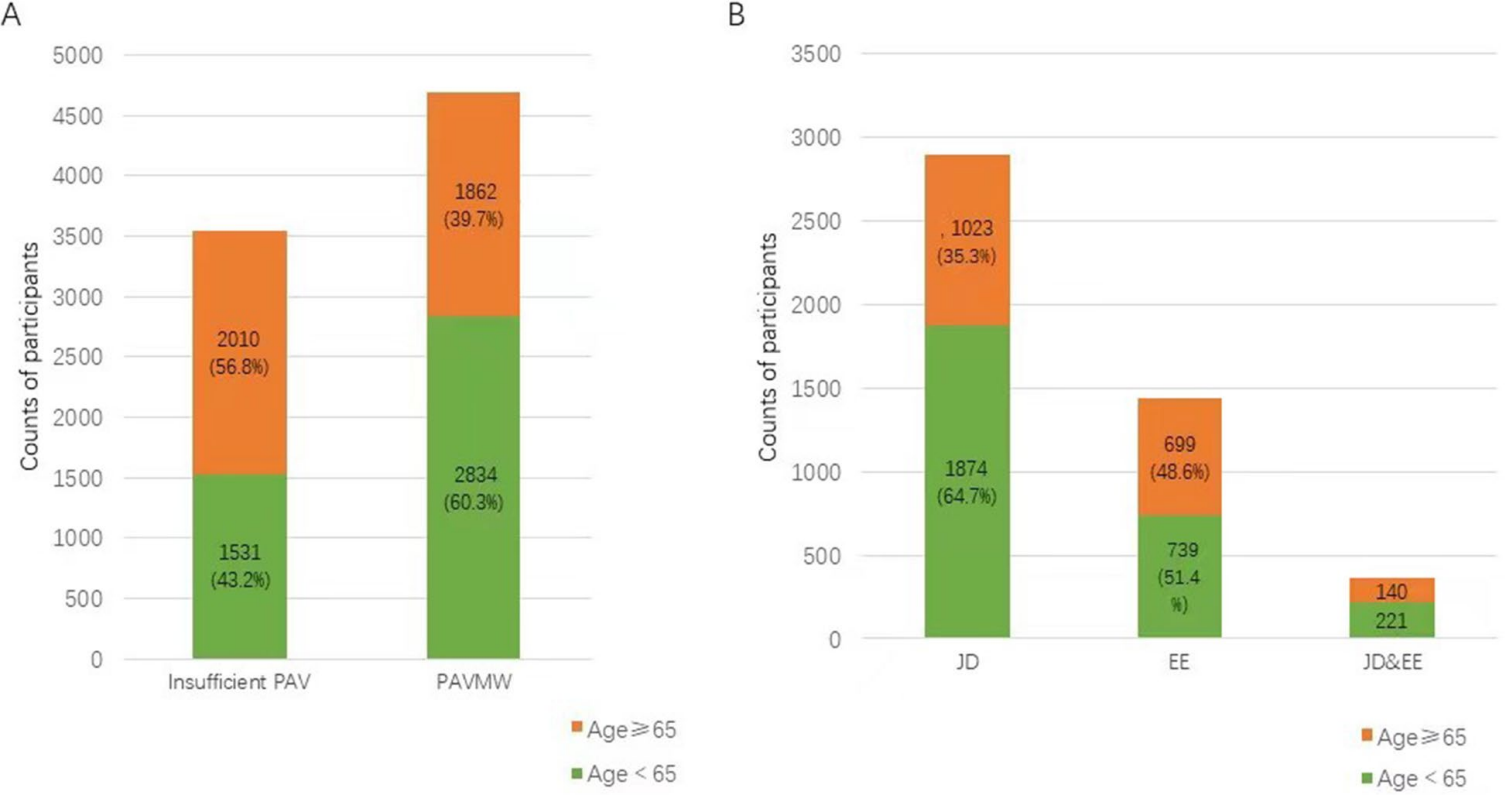
Distribution of participants with different physical activity levels, purposes, and ages. Part A: Distribution of participants with different physical activity levels and ages: Insufficient PAV: the physical activity volume does not meet the WHO’s recommendations; PAVMW: the physical activity volume meets the WHO’s recommendations. Part B: Distribution of participants meeting the WHO’s recommendations with different physical activity purposes and ages; JDs: job demands; EE: entertainment or exercise; JDs&EE: job demands and entertainment or exercise

Among the 8237 eligible participants, 43% (*n* = 3541) of the participants’ PAVs were less than 150 min of MPAV per week, and 57% (*n* = 4696) of the participants met the WHO recommendations for PAV per week. Compared with the participants who did not meet the WHO’s recommendations, those who met the WHO’s recommendations were more likely to be middle-aged (< 65), be male, have a normal BMI (≥18.5 and < 25), have a high school education or below, be former drinkers, never have smoked, and have no chronic diseases. Table 2 shows the baseline characteristics of the study participants. There were statistically significant differences in each baseline characteristic between the two groups (all<0.05). Figure 1 shows the age distribution of the participants with different levels of PAV. Elderly individuals accounted for a high proportion of the population with insufficient PA levels. Middle-aged people accounted for a high proportion of people who met the WHO recommendations; however, the proportion of elderly people who performed PA for entertainment and exercise purposes was higher than that of elderly people who performed PA for job purposes.

**Table 2:** Baseline characteristics

Characteristics	Insufficient PA (***n*** = 3541)	PA meeting the WHO’s recommendations (***n*** = 4696)	***P*** value
Age (years):			
45–64(4365)	1531(43.2)	2834(60.3)	*P* < 0.001
≥ 65(3872)	2010(56.8)	1862(39.7)	
Sex:			
Women (4443)	1634(46.1)	2160(46.0)	*P* < 0.05
Man(3794)	1907(53.9)	2536(54.0)	
BMI:			
<18.5(514)	271(7.7)	243(5.2)	*P* < 0.01
18. ≤ and<25(5274)	2216(62.6)	3058(65.1)	
25 ≤ and < 30(2093)	880(24.9)	1213(25.8)	
≥ 30(356)	174(4.9)	182(3.9)	
Education:			
≤ Highschool(7917)	3381(95.5)	4536(96.6)	*P* < 0.01
≥ College(320)	160(4.5)	160(3.4)	
Drinking status:			
Current(2124)	861(24.3)	1263(26.9)	*P* < 0.001
Former(694)	273(7.7)	421(9.0)	
Never(5419)	2407(68.0)	3012(64.1)	
Smoking status:			
Current(2562)	1116(31.5)	1446(30.8)	*P* < 0.001
Former(594)	271(7.7)	323(6.9)	
Never(5081)	2154(60.8)	2927(62.3)	
Other chronic conditions:			
Hypertension:			
Untreated(395)	170(4.8)	225(4.8)	*P* < 0.001
Treated(1074)	562(15.9)	512(10.9)	
None(6768)	2809(79.3)	3959(84.3)	
Hyperlipidaemia:			
Untreated(257)	115(3.2)	142(3.0)	*P* < 0.01
Treated(273)	150(4.2)	123(2.6)	
None(7707)	3276(92.5)	4431(94.4)	
Diabetes:			
Untreated(109)	50(1.4)	59(1.3)	*P* < 0.001
Treated(216)	133(3.8)	83(1.8)	
None(7912)	3358(94.8)	4554(97.0)	
Lung disease:			
Untreated(275)	130(3.7)	145(3.1)	*P* < 0.01
Treated(338)	161(4.5)	177(3.8)	
None(7624)	3250(91.8)	4374(93.1)

### Physical activity and heart disease incidence rate

During the seven-year follow-up from 2011 to 2018 (*n* = 8237), 6.5% (*n* = 534) of the participants suffered from heart disease. Table 3 shows the correlation between different PAVs and the risk of heart disease. In Model 1 and Model 2, compared with the participants who did not meet the WHO’s recommended PAV, those who did meet the WHO’s recommended PAV reduced their risk of heart disease by 21% (Model 1: HR = 0.79, 95% CI 0.66 to 0.93; Model 2: HR = 0.79, 95% CI 0.67 to 0.94). In the fully adjusted model (Model 3), compared with the participants who did not meet the WHO’s recommended PAV, those who did meet the WHO’s recommended PAV reduced their risk of heart disease by 20% (HR = 0.80, 95% CI 0.68 to 0.96).

**Table 3:** Association between study participants engaging in physical activities according to the WHO physical activity recommendations and heart disease morbidity

Variables	Model 1	Model 2	Model 3
Insufficient PA	ref	ref	ref
PA meeting the WHO’s recommendations	0.79(0.66–0.93)	0.79(0.67–0.94)	0.80(0.68–0.96)
For work	0.75 (0.61–0.92)**	0.77 (0.63–0.95)*	0.79 (0.65–0.97)*
For leisure	0.92 (0.73–1.16)	0.90 (0.72–1.13)	0.90(0.72–1.13)
For both work and leisure	0.44 (0.25–0.79)**	0.45 (0.25,0.81)**	0.46 (0.26–0.83)*

*PA* Physical activity, *HD* Heart disease. Values are hazard ratios (95% confidence intervals)

Model 1: Adjusted for sex and age

Model 2: Model 1 + education level, body mass index, smoking status, and drinking status

Model 3: Model 2 + chronic conditions

### Subgroup analysis

**Fig. 2:**
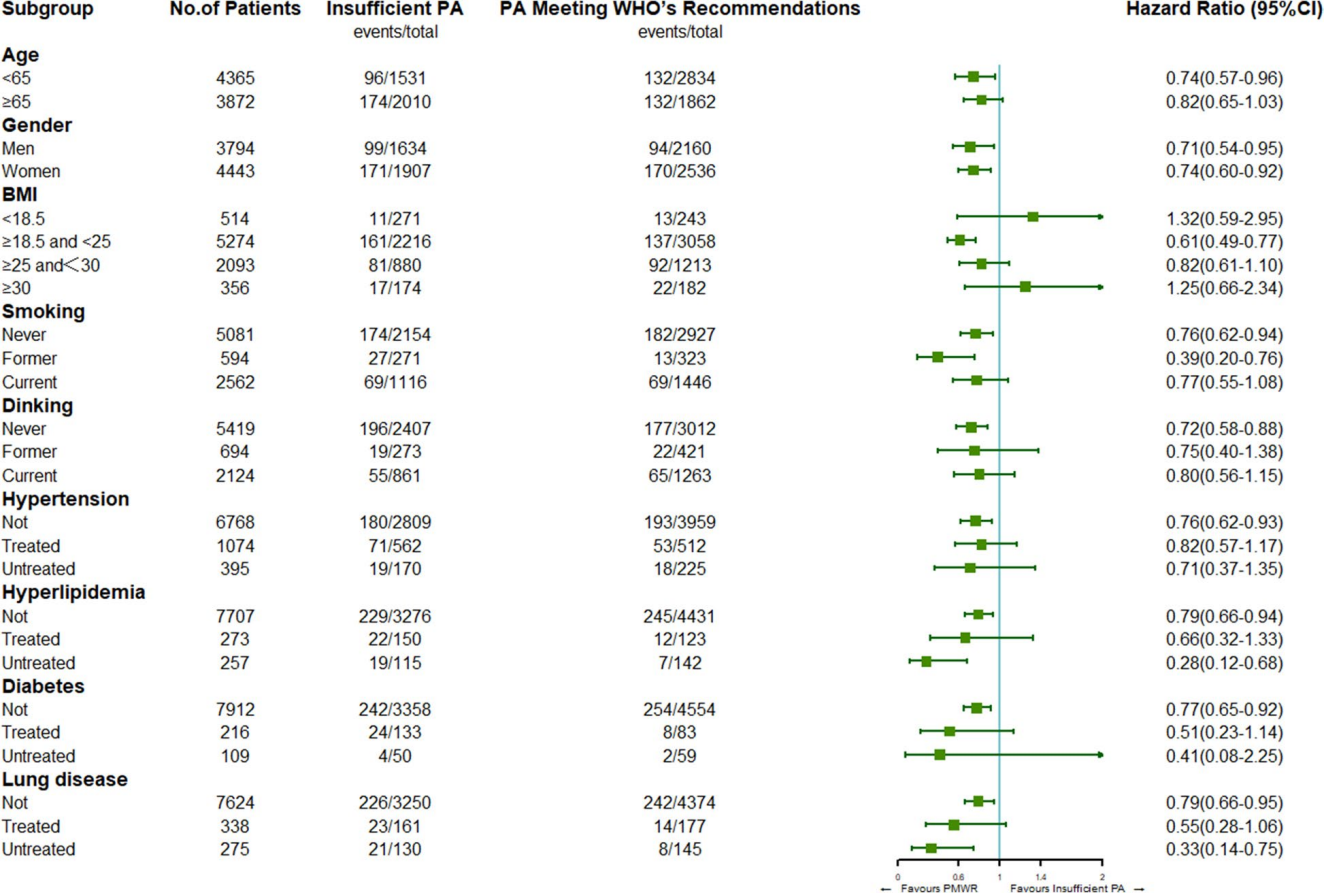
Subgroup analyses of the association between study participants engaging in physical activities according to the WHO physical activity recommendations and heart disease incidence rate. PMWR: PAV meeting the WHO’s recommendations

All potential covariates (age, sex, education level, BMI, smoking status, drinking status, and chronic diseases) with the PAV had no significant interactive effects on the incidence rate of heart disease. Figure 2 shows the results of the subgroup analysis. Male participants who met the WHO’s recommendations for PAV had a lower risk of heart disease (HR = 0.71) than female participants (HR = 0.74). In addition, when the participants met the WHO’s recommendations for PAV, the risk of heart disease in middle-aged people decreased by 26%; the risk in those in the normal BMI range (≥18.5 and < 25) decreased by 49%; the risk in participants without hypertension decreased by 24%; the risk in participants without hyperlipidaemia decreased by 21%; and the risk in participants without lung disease decreased by 21%. It is worth noting that when the participants met the WHO’s recommendations for PAV, the risk of heart disease among untreated participants with hyperlipidaemia or lung disease was significantly reduced, with HRs of 0.28 and 0.33, respectively.

### Sensitivity analysis

We conducted a sensitivity analysis of the data to confirm the results of the study. The risk of heart disease in those who met the WHO’s recommendations decreased by 19% (HR = 0.81, 95% CI 0.73 to 0.91) compared with those who did not meet the recommendations, which was similar to the results of the previous study that deleted the missing data.

## Discussion

The results of this study show that the WHO’s recommendations for PAV can help Chinese middle-aged and older people reduce their risk of heart disease. Among the people meeting the WHO recommendations for PAV, men had a lower risk of heart disease than women and the risk of heart disease was reduced in middle-aged people and participants with normal BMI. In addition, participants without hyperlipidaemia and lung disease had a lower risk of heart disease. It should be noted that the participants with untreated hyperlipidaemia and untreated lung disease also showed a decrease in the risk of heart disease.

Compared with the decline in heart disease morbidity in the United States, the United Kingdom and Central and Eastern Europe in the past 30 years [21–23], rapid urbanization in the past few decades [24] has led to a continuous increase in the morbidity of heart disease in China (for example, the morbidity increased by approximately 15% from 1990 to 2016), which is expected to continue to rise in the next few decades [25]. At the same time, the cost of hospitalization caused by heart disease is also increasing rapidly [5], which may further expand China’s social and economic burdens. In this study, only 57% of the participants met the WHO’s recommendations for PA, which reflects that more middle-aged and older people in China still lack physical activity. Therefore, the Chinese government and administrative departments should formulate corresponding measures as soon as possible to enable middle-aged and older people to meet the WHO’s recommendations for PA to reduce the incidence rate of heart disease and to reduce the damage to society and the economy that is caused by heart disease.

This study found that the risk of heart disease decreased significantly in participants who performed physical activity for the purpose of work, but there was no change in the risk of heart disease among participants who performed physical activity for leisure purposes. This is different from the previous view of the benefits of physical activity at work and for leisure purposes, but some of the physical activity benefits may be work-related [26] basically consistent. However, some scholars believe that higher leisure physical activity can reduce the risk of all-cause death, and higher professional physical activity will increase the risk of heart disease and other diseases [27–30]. This is not consistent with our results, and we suspect that the reasons for the above differences may be as follows: First, China is still a developing country, and many jobs are still dominated by physical activities, while in developed countries, many employees are engaged in mental work, and the proportion of light physical activities is higher [31, 32]. Therefore, compared with developed countries, China’s middle-aged and elderly population groups can better meet the physical activity standards recommended by the WHO. Second, there is a lack of professional leisure sports instructors in China, so the methods of exercise in leisure activities for the elderly population cannot achieve the best results because of the lack of guidance. This may reduce the role of physical activities with leisure as the main purpose in preventing heart disease. Third, the education level of the groups we included was low. The research shows that the lack of leisure sports activities is more common among people with a low education level. The main reason is that a low understanding of the importance of leisure sports leads to a lack of participation in leisure sports activities [33–35]. Therefore, for the middle-aged and elderly groups in China, the contribution of occupational physical activity to total physical activity is higher than that of leisure physical activity. This may lead to a significant reduction in the risk of heart disease among participants performing occupational physical activity, but there is no change in the risk of heart disease among participants performing leisure sports activities. However, the above explanation still needs to be further verified in future high-quality research.

Relevant studies have explained that there may be significant differences between men and women in the correlation between physical activity and heart disease and the benefits of physical activity in reducing the risk of coronary heart disease due to sex-specific factors (including hormones) [36]. In addition, many women with heart disease are not diagnosed because many of their symptoms (such as jaw pain, fatigue, nausea and indigestion) are not detected or are misdiagnosed [37]. We also found that in the participants who met the WHO’s recommendations for PA, the risk of heart disease in those who were middle-aged was reduced, which is consistent with the previous finding that the physical activity of middle-aged people is negatively correlated with heart disease [38]. This study did not find that the WHO’s recommendations for PA had a significant benefit in reducing the risk of heart disease in the elderly population, which may be because elderly individuals are mostly retired; as we mentioned, their modes of physical activity are mostly leisure exercises without correct guidance. This study found that the participants who met the WHO’s recommendations for PA and had a normal BMI had a lower risk of heart disease, but this was not observed for obese or overweight participants. At present, overweight and obesity have become a global epidemic. According to the National Physique Monitoring Report in 2014, the prevalence rates of overweight and obesity among the elderly population in China in 2014 were 41.6 and 13.9%, respectively, which was 1.8 and 0.9% higher than those in 2010 [39]. Previous studies have shown that an increase in BMI leads to a significant increase in the prevalence of heart disease [40], which may offset the benefits of PA, even if the WHO’s recommendations are met. In addition, studies have shown that there is a complex association among smoking, alcohol consumption and cardiovascular disease; for example, excessive drinking every day increases the risk of cardiovascular disease [41], and smoking only one cigarette per day also increases the risk of cardiovascular disease [42], which may offset the benefits of the amount of exercise recommended by the WHO. Therefore, while meeting the WHO’s recommendations for PA, Chinese middle-aged and older people should reasonably control their weight, smoking and drinking, which may play an effective role in preventing heart disease.

In the participants who met the WHO’s recommendations for PA, we did not find that those with hypertension, hyperlipidaemia and lung disease had significant benefits in reducing the risk of heart disease. Hypertension is an important risk factor for heart disease, affecting more than 1 billion people around the world [43, 44]. Globally, although there are guidelines for treatment, only one-third of adults reach the target blood pressure [45], and patients usually need to prevent sequelae such as heart disease through the use of antihypertensive drugs [46, 47]. Diabetes has become a worldwide epidemic disease that is characterized by sustained hyperglycaemia caused by the improper function or diminished secretion of insulin [48]. Persistent hyperglycaemia can lead to serious complications, such as heart disease [49]. Hyperlipidaemia is one of the risk factors for cardiovascular disease and increases the risk of nonischaemic heart failure. In addition, hyperlipidaemia indirectly affects cardiac function by promoting the development of atherosclerosis and directly affects cardiac systolic function and the cardiac electrophysiological response, which may be related to subsequent systemic oxidative stress along with the gradual accumulation of cardiac lipids, a proinflammatory state, and mitochondrial dysfunction [37]. Some studies have pointed out that many lung diseases at onset or after treatment may induce the sequelae of increasing right ventricular afterload, and right ventricular contraction can be prolonged due to the increase in afterload, eventually leading to the occurrence of heart disease [50]. Regardless, the occurrence of hypertension, hyperlipidaemia and lung disease may offset the benefits of physical activity. Notably, our results showed that participants with untreated hyperlipidaemia and untreated lung disease could have a significant reduction in the risk of heart disease when they met the WHO’s recommendations for PA. Although this result may be related to the inadequate sample size of the study, it can suggest that patients with hyperlipidaemia and lung disease still have the benefit of preventing heart disease by following the WHO’s recommendations for PA before receiving standard treatment or refusing drug treatment.

The strengths of this study are as follows: first, this study was a cohort study with a sample that was followed up for 7 years; second, the data source database of this study has been referenced by many high-quality research institutes. This study used a large representative sample from China, so these results can be extended to the whole Chinese population. Finally, we considered and adjusted 10 confounding factors, including personal variables, health-related variables, lifestyle variables and chronic diseases. The limitations of this study are as follows: first, there may be some deviation in the data because the research indicators were measured by self-report questionnaires. Second, although we dealt with the missing data, it may still have a certain impact on the results of this study. Third, there is a lack of questions regarding diet on the questionnaire, so there was no analysis of diet in our covariates, which may have a certain impact on the research results. Finally, the questions on the CHARLS questionnaire are not accurate enough; for example, the measurement span of PA duration is too wide.

## Conclusions

This study revealed that meeting the WHO’s recommendations for physical activity volume can reduce the risk of heart disease in middle-aged and older people in China and can also effectively prevent heart disease for people with some common chronic diseases, such as hyperlipidaemia and lung disease. The results showed that physical activity for leisure and exercise had a lower preventive effect on heart disease than physical activity for a job, which may be related to the inappropriate leisure and exercise methods of the participants.

## Data Availability

The questionnaire data supporting the conclusions of this article is available at http://charls.pku.edu.cn/.

## References

[1] Hu SS, Kong LZ, Gao RL, Zhu ML, Wang W, Wang YJ, Wu ZS, Chen WW, Liu MB (2012). Outline of the report on cardiovascular disease in China, 2010. Biomed Environ Sci.

[2] Suinesiaputra A, McCulloch AD, Nash MP, Pontre B, Young AA (2016). Cardiac image modelling: breadth and depth in heart disease. Med Image Anal.

[3] Sofi F, Capalbo A, Cesari F, Abbate R, Gensini GF (2008). Physical activity during leisure time and primary prevention of coronary heart disease: an updated meta-analysis of cohort studies. Eur J Cardiovasc Prev Rehabil.

[4] K. S: The Global Economic Burden of Noncommunicable Diseases. *F, 2012 [C]* 2012.

[5] Zhang J, Lu N (2021). The association between childhood conditions and heart disease among middle-aged and older population in China: a life course perspective. BMC Geriatr.

[6] Liu J, Ma C (2019). Current state of cardiovascular research in China. Nat Rev Cardiol.

[7] Chen Z, Peto R, Zhou M, Iona A, Smith M, Yang L, Guo Y, Chen Y, Bian Z, Lancaster G (2015). Contrasting male and female trends in tobacco-attributed mortality in China: evidence from successive nationwide prospective cohort studies. Lancet.

[8] Lv J, Yu C, Guo Y, Bian Z, Yang L, Chen Y, Tang X, Zhang W, Qian Y, Huang Y (2017). Adherence to healthy lifestyle and cardiovascular diseases in the Chinese population. J Am Coll Cardiol.

[9] National Center for chronic disease P, health promotion office on S, health: reports of the surgeon general. In: The health consequences of smoking—50 years of Progress: a report of the surgeon general. Atlanta (GA): Centers for Disease Control and Prevention (US); 2014.

[10] Joseph P, Leong D, McKee M, Anand SS, Schwalm JD, Teo K, Mente A, Yusuf S (2017). Reducing the global burden of cardiovascular disease, part 1: the epidemiology and risk factors. Circ Res.

[11] Manson JE, Greenland P, LaCroix AZ, Stefanick ML, Mouton CP, Oberman A, Perri MG, Sheps DS, Pettinger MB, Siscovick DS (2002). Walking compared with vigorous exercise for the prevention of cardiovascular events in women. N Engl J Med.

[12] Wilson MG, Ellison GM, Cable NT (2016). Basic science behind the cardiovascular benefits of exercise. Br J Sports Med.

[13] Zhao D, Liu J, Wang M, Zhang X, Zhou M (2019). Epidemiology of cardiovascular disease in China: current features and implications. Nat Rev Cardiol.

[14] Fiuza-Luces C, Santos-Lozano A, Joyner M, Carrera-Bastos P, Picazo O, Zugaza JL, Izquierdo M, Ruilope LM, Lucia A (2018). Exercise benefits in cardiovascular disease: beyond attenuation of traditional risk factors. Nat Rev Cardiol.

[15] Vega RB, Konhilas JP, Kelly DP, Leinwand LA (2017). Molecular mechanisms underlying cardiac adaptation to exercise. Cell Metab.

[16] Wang S, Ren J (2018). Obesity paradox in aging: from prevalence to pathophysiology. Prog Cardiovasc Dis.

[17] Seo DY, Kwak HB, Kim AH, Park SH, Heo JW, Kim HK, Ko JR, Lee SJ, Bang HS, Sim JW (2020). Cardiac adaptation to exercise training in health and disease. Pflugers Arch.

[18] Bull FC, Al-Ansari SS, Biddle S, Borodulin K, Buman MP, Cardon G, Carty C, Chaput JP, Chastin S, Chou R (2020). World Health Organization 2020 guidelines on physical activity and sedentary behaviour. Br J Sports Med.

[19] Gersh BJ, Sliwa K, Mayosi BM, Yusuf S (2010). Novel therapeutic concepts: the epidemic of cardiovascular disease in the developing world: global implications. Eur Heart J.

[20] Zeng Z, Bian Y, Cui Y, Yang D, Wang Y, Yu C. Physical activity dimensions and its association with risk of diabetes in middle and older aged Chinese people. Int J Environ Res Public Health. 2020;17(21). 10.3390/ijerph17217803 PMC7663282 33113802

[21] Benjamin EJ, Muntner P, Alonso A, Bittencourt MS, Callaway CW, Carson AP, Chamberlain AM, Chang AR, Cheng S, Das SR (2019). Heart disease and stroke Statistics-2019 update: a report from the American Heart Association. Circulation.

[22] Ford ES, Roger VL, Dunlay SM, Go AS, Rosamond WD (2014). Challenges of ascertaining national trends in the incidence of coronary heart disease in the United States. J Am Heart Assoc.

[23] Townsend N, Wilson L, Bhatnagar P, Wickramasinghe K, Rayner M, Nichols M (2016). Cardiovascular disease in Europe: epidemiological update 2016. Eur Heart J.

[24] Zhang Q, Zhao D, Xie W, Xie X, Guo M, Wang M, Wang W, Liu W, Liu J (2016). Recent trends in hospitalization for acute myocardial infarction in Beijing: increasing overall burden and a transition from ST-segment elevation to non-ST-segment elevation myocardial infarction in a population-based study. Medicine.

[25] Liu S, Li Y, Zeng X, Wang H, Yin P, Wang L, Liu Y, Liu J, Qi J, Ran S (2019). Burden of cardiovascular diseases in China, 1990-2016: findings from the 2016 global burden of disease study. JAMA Cardiol.

[26] Donahue RP, Abbott RD, Reed DM, Yano K (1988). Physical activity and coronary heart disease in middle-aged and elderly men: the Honolulu heart program. Am J Public Health.

[27] Dalene KE, Tarp J, Selmer RM, Ariansen IKH, Nystad W, Coenen P, Anderssen SA, Steene-Johannessen J, Ekelund U (2021). Occupational physical activity and longevity in working men and women in Norway: a prospective cohort study. Lancet Public Health.

[28] Holtermann A, Krause N, van der Beek AJ, Straker L (2018). The physical activity paradox: six reasons why occupational physical activity (OPA) does not confer the cardiovascular health benefits that leisure time physical activity does. Br J Sports Med.

[29] Holtermann A, Schnohr P, Nordestgaard BG, Marott JL (2021). The physical activity paradox in cardiovascular disease and all-cause mortality: the contemporary Copenhagen general population study with 104 046 adults. Eur Heart J.

[30] Smith P, Ma H, Glazier RH, Gilbert-Ouimet M, Mustard C (2018). The relationship between occupational standing and sitting and incident heart disease over a 12-year period in Ontario, Canada. Am J Epidemiol.

[31] Brownson RC, Boehmer TK, Luke DA (2005). Declining rates of physical activity in the United States: what are the contributors?. Annu Rev Public Health.

[32] Church TS, Thomas DM, Tudor-Locke C, Katzmarzyk PT, Earnest CP, Rodarte RQ, Martin CK, Blair SN, Bouchard C (2011). Trends over 5 decades in U.S. occupation-related physical activity and their associations with obesity. PLoS One.

[33] Droomers M, Schrijvers CT, van de Mheen H, Mackenbach JP (1988). Educational differences in leisure-time physical inactivity: a descriptive and explanatory study. Soc Sci Med.

[34] Rupps E, Haenle MM, Steinacker J, Mason RA, Oeztuerk S, Steiner R, Kratzer W (2012). Physical exercise in southern Germany: a cross-sectional study of an urban population. BMJ Open.

[35] Coday M, Klesges LM, Garrison RJ, Johnson KC, O'Toole M, Morris GS (2002). Health opportunities with physical exercise (HOPE): social contextual interventions to reduce sedentary behavior in urban settings. Health Educ Res.

[36] Batty GD (2002). Physical activity and coronary heart disease in older adults. A systematic review of epidemiological studies. Eur J Pub Health.

[37] Gorgos D. Women's cardiac centers focus on the gender differences of heart disease.(WOMEN'S HEALTH UPDATE). Dermatol Nurs. 2006.

[38] Yano K, Reed DM, McGee DL (1984). Ten-year incidence of coronary heart disease in the Honolulu heart program. Relationship to biologic and lifestyle characteristics. Am J Epidemiol.

[39] Hou Q, Guan Y, Yu W, Liu X, Wu L, Xiao M, Lü Y (2019). Associations between obesity and cognitive impairment in the Chinese elderly: an observational study. Clin Interv Aging.

[40] Lamon-Fava S, Wilson PW, Schaefer EJ (1996). Impact of body mass index on coronary heart disease risk factors in men and women. The Framingham offspring study. Arterioscler Thromb Vasc Biol.

[41] Piano MR (2017). Alcohol's effects on the cardiovascular system. Alcohol Res.

[42] Kondo T, Nakano Y, Adachi S, Murohara T (2019). Effects of tobacco smoking on cardiovascular disease. Circul J.

[43] Mozaffarian D, Benjamin EJ, Go AS, Arnett DK, Blaha MJ, Cushman M, de Ferranti S, Després JP, Fullerton HJ, Howard VJ (2015). Heart disease and stroke statistics--2015 update: a report from the American Heart Association. Circulation.

[44] Arwood MJ, Cavallari LH, Duarte JD (2015). Pharmacogenomics of hypertension and heart disease. Curr Hypertens Rep.

[45] Thoenes M, Neuberger HR, Volpe M, Khan BV, Kirch W, Böhm M (2010). Antihypertensive drug therapy and blood pressure control in men and women: an international perspective. J Hum Hypertens.

[46] Hsu CY, McCulloch CE, Darbinian J, Go AS, Iribarren C (2005). Elevated blood pressure and risk of end-stage renal disease in subjects without baseline kidney disease. Arch Intern Med.

[47] Lawes CM, Vander Hoorn S, Rodgers A (2008). Global burden of blood-pressure-related disease, 2001. Lancet.

[48] Wojciechowska J, Krajewski W, Bolanowski M, Kręcicki T, Zatoński T (2016). Diabetes and Cancer: a review of current knowledge. Exp Clin Endocrinol Diabetes.

[49] Clodi M, Saely C, Hoppichler F, Resl M, Steinwender C, Eber B (2016). Diabetes mellitus, coronary artery disease and heart disease. Wien Klin Wochenschr.

[50] Fallon CK (2019). Husbands' hearts and Women's health: gender, age, and heart disease in twentieth-century America. Bull Hist Med.

